# The effect of distributive justice climate on virtual team performance: A moderated mediation model

**DOI:** 10.3389/fpsyg.2022.950581

**Published:** 2022-08-02

**Authors:** Xuan Yu, Bin He, Meilin Liu, Ai Wang, Yue Yuan

**Affiliations:** ^1^School of Economics and Management, Southwest Petroleum University, Chengdu, China; ^2^School of State Governance, Southwest University, Chongqing, China; ^3^Antai College of Economics and Management, Shanghai Jiao Tong University, Shanghai, China; ^4^School of Foreign Language Department, Civil Aviation Flight University of China, Chengdu, China

**Keywords:** distributive justice climate, high-quality relationships, team proactive personality, virtual team performance, social interdependence theory, proactive personality theory

## Abstract

Based on the social interdependence theory, we proposed that the distributive justice climate affects virtual team performance *via* high-quality relationships, and then we investigated the boundary effect of team proactive personality. The data used in this study were collected in China, including 327 virtual team members that belonged to 75 teams. The following results are obtained: (1) Distributive justice climate and high-quality relationships have significant positive effects on virtual team performance. (2) High-quality relationships mediate the relationship between the distributive justice climate and virtual team performance. (3) Team proactive personality strengthens the direct effect of the distributive justice climate on high-quality relationships. (4) Team proactive personality strengthens the indirect effect of the distributive justice climate on virtual team performance through high-quality relationships. These empirical results have important theoretical significance for team climate construction, personnel selection, and team performance promotion.

## Introduction

Talent collection, information circulation, knowledge sharing, and organizational structure flexibility are no longer difficult problems because of the development of modern network communication technology; consequently, an important opportunity to develop and manage virtual teams is provided to knowledge-based organizations (Kirkman et al., 2004). A virtual team is a typical task team that relies on the support of modern network communication media to overcome obstacles across time, space, or organizational boundaries, and ultimately achieve a common mission and purpose (Griffith and Neale, 2003). Over the past few decades, the number of organizations adopting virtual teams has increased tremendously, and this trend is expected to continue (Dulebohn and Hoch, 2017). Although virtual teams can effectively respond collectively to a rapidly changing environment and create greater value for an organization, they are facing greater challenges due to market opportunity uncertainty and organizational complexity, and only virtual teams with concentrated high-quality resources can achieve better performance for an organization (Workman et al., 2003). Therefore, improving virtual team performance is critical in ensuring that high-tech enterprises achieve victory in fierce market competitions.

The antecedents of virtual team performance have always been the focus of scholars. Previous studies have discussed organizational situational factors (e.g., cultural climate, time pressure, and reward structure), group factors (e.g., group scale, group similarity, group composition, and team cohesion), task factors (e.g., task type and task complexity), and technical factors (e.g., choice of communication tools) (Lurey and Raisinghani, 2001). Among organizational situation factors, team atmosphere, as a sharing perception of team members about their working environment, is typically regarded as an important input variable that affects team performance; its effect on virtual team performance should not be underestimated (Anderson and West, 1998; Mathieu et al., 2008). A distributive justice climate describes the common views of members on the fair distribution of organizational resources and rewards (Whitman et al., 2012). Existing studies have found that a distributive justice climate exerts significant positive effects on team performance in traditional physical teams (Lester et al., 2002; Lipponen and Wisse, 2010; Zhou and Li, 2015). Zhou and Li (2015) proposed that industrial relation climate mediates the relationship between the distributive justice climate and employees’ job performance, identifying the internal mechanisms of the effect of the distributive justice climate on job performance.

However, studies on the relationship between the distributive justice climate and virtual team performance have focused on theoretical exploration stages and literature reviews. Empirical tests should be conducted to draw more scientific conclusion and promote the development of existing studies; virtual team members must strengthen high-quality connections to achieve their common mission and purpose successfully (Stephens et al., 2011). Virtual team members involved in the current study belong to the same organization and have a certain social basis. They use electronic communication tools to communicate and complete tasks due to different working places or asynchronous working hours. The establishment and maintenance of relationships are not only the basis of virtual team operation, but also important premises of virtual team management (Breuer et al., 2016). Considering that the environment faced by a virtual team is characterized by task complexity, knowledge intensity, and task interdependence, relational coordination should be highly critical, and the primary purpose of high-quality relationships is to accelerate relationship coordination (Algoe, 2020). High-quality relationships refer to relationships generated by the interaction among team members in the workplace to achieve team goals; they include three dimensions, i.e., shared goals, shared knowledge, and mutual respect (Gittell, 2002), which are not only focused on interpersonal communication. In accordance with social interdependence theory, social interdependence will exist when the results of individuals and teams are influenced by their actions and those of others (Johnson and Johnson, 2005; Johnson, 2009). Therefore, this study inferred that when individuals in virtual teams perceive a distributive justice climate, they will interact with other members, establish high-quality relationships, and subsequently improve virtual team performance. The current study introduced high-quality relationships as the mediator of the distributive justice climate and virtual team performance. Previous studies have focused on industrial relations climate in traditional physical teams (Zhou and Li, 2015), and this study opens the black box between the distributive justice climate and team performance in virtual teams from the perspective of social interaction, and thus, it is different from previous studies.

Virtual team members are scattered in different areas, leading to the lack of behavioral control of virtual team managers and the loss of task timeliness. Virtual team members have less face-to-face and informal communication, and are prone to isolation and negative emotions, affecting their performance (Purvanova, 2014). In accordance with social interdependence theory, different members are influenced by their own actions and those of others (Johnson, 2009). Members with active personality traits are more inclined to understand the surrounding environment and exhibit proactive behavior (Seibert et al., 2001). They not only build effective social networks, but also work more actively (Thompson, 2005; Grant and Ashford, 2008). Although proactive personality is typically determined at the individual level, some scholars have proposed team proactive personality (Wang et al., 2017). The feasibility and necessity of conducting proactive personality research at the team level have also demonstrated (Grant et al., 2011; Bradley et al., 2013). A team’s proactive personality represents a collective tendency to initiate problem solving, self-management, and self-improvement activities that promote team motivation (Williams et al., 2010). Accordingly, the current study infers that when a distributive justice climate is perceived by team members with higher proactive personalities, these members have common views on the fair distribution of organizational resources and rewards (Whitman et al., 2012). They are more likely to demonstrate a collective tendency to initiate problem solving and self-improvement activities, thus improving virtual team performance through high-quality relationships.

On this basis, we propose that a team proactive personality strengthens the direct effect of a distributive justice climate on high-quality relationships and its indirect effect on virtual team performance through high-quality relationships.

### Theory and hypothesis development

#### Distributive justice climate and virtual team performance

Previous studies have used traditional physical teams as research objects, basically confirming that a distributive justice climate exerts a positive effect on their performance (Lipponen and Wisse, 2010; Whitman et al., 2012). In contrast with traditional physical teams, virtual teams do not have fixed offices, and members rely on modern network communication technology to cooperate with one another to complete their tasks (Griffith and Neale, 2003; Wang et al., 2022). Existing studies have regarded climate as the key issue for virtual teams (Martins et al., 2004). A distributive justice climate may be critical for virtual teams; it is conducive to solving many problems caused by remote communication and collaboration among team members, and thus, it is conducive to improving virtual team performance.

On the one hand, some studies have pointed out that the primary problem of virtual team performance management is dealing with resource allocation (Kimble, 2011). Maximum value will only be created by providing each member with the right incentives (Kirkman et al., 2004). Employees may interpret distributive justice climate as the guarantee to organizational economic outcomes (e.g., money) (Colquitt et al., 2013). Moreover, distributive justice climate is regarded as an indicator that shows that organizations focus on the contributions of individuals and will treat them in a fair and equitable manner, encouraging employees to invest time and energy to accomplish tasks and achieve organizational goals (Zhou and Li, 2015), ultimately helping improve virtual team performance.

On the other hand, virtual teams are frequently dynamic, knowledge-intensive, and task interdependence situations; thus, virtual teams require members to cooperate with one another to accomplish tasks and achieve organizational goals (Hoch and Kozlowski, 2014). Virtual team performance is the collection of individual performance, and improvement in team performance requires each team member to complement one another (Griffith and Neale, 2003). Therefore, the whole team may be strengthened only when each virtual team member is firmly connected, and connectivity is constructed and strengthened (Dutton and Heaphy, 2003). The current study concludes that in situations characterized by task complexity, knowledge intensity, and task interdependence, distributive justice climate, as the shared perceptions of virtual team members regarding their workplace environment, plays an “adhesive” role by bringing together members scattered in different regions, time zones, and professional backgrounds, encouraging them to participate in remote cooperation in virtual space, promoting the performance of each team member, and then surpassing personal factors that affect virtual team performance. Therefore, the following hypothesis is presented:

*Hypothesis 1:* Distributive justice climate is positively related to virtual team performance.

#### High-quality relationships and virtual team performance

High-quality relationships are important representations of the quality of organizational interpersonal relationships, which reflect the interactive process of communication to accomplish tasks; it includes three dimensions: shared goals, shared knowledge, and mutual respect (Gittell, 2002). The positive effect of high-quality relationships on virtual team performance is primarily reflected in the following three aspects.

First, shared goals are conducive to improving virtual team performance. Significant differences exist among virtual team members in terms of culture, professional background, and time orientation, easily leading to conflicts among individuals, individual goals, and team goals (Breuer et al., 2016). Shared goals may unify the values of team members to a certain extent, resulting in cooperation toward achieving individual and team goals, forming a consistent vision and organizational commitment, reducing the probability of conflict among members, and helping members with the division of labor to improve virtual team performance (Algoe, 2020).

Second, shared knowledge is conducive to improving virtual team performance. Virtual teams have high task interdependence and rely heavily on collaborative effort; hence, team performance is strongly influenced by the ability to deal with problems and learn (Hoch and Kozlowski, 2014). Shared knowledge among members helps improve the integration of various abilities, knowledge, and resources; promotes the effective coordination of tasks; and then accomplishes those complex tasks beyond individual cognitive ability; that is, shared knowledge may enhance the information processing and learning ability of an organization to improve virtual team performance (Den Hooff and De Ridder, 2004; Brueller and Carmeli, 2011).

Third, mutual respect is conducive to improving virtual team performance. It may improve the quality of communication among team members who play different roles in labor division, encourage members to pay attention to the value of others in the process of mutual cooperation, bring interpersonal distance closer, and deepen mutual trust (Carmeli and Gittell, 2009); mutual trust among virtual team members may promote virtual team performance (Jarvenpaa et al., 1998). High-quality relationships act as important potential buffers against threats to improve virtual team performance (Schermuly and Meyer, 2016). Therefore, the following hypothesis is proposed:

*Hypothesis 2:* High-quality relationships are positively related to virtual team performance.

#### Mediating effect of high-quality relationships

This study proposes that a distributive justice climate facilitates the development of high-quality relationships in virtual teams. First, a distributive justice climate is conducive to helping members share goals. It is a form of personal subjective feeling. If members feel that they are treated fairly by their organization, then they are more willing to share its values and achieve organizational objectives (Whitman et al., 2012). Second, distributive justice climate is conducive to helping members share knowledge. Studies have shown that a fair distribution of organizational resources and rewards is the basis for establishing and maintaining a distributive justice climate, which can encourage and promote employees’ knowledge-sharing behavior (Bartol and Srivastava, 2002). Third, a distributive justice climate is conducive to building mutual respect among members. Previous studies have found that distributive justice climate may promote the formation of long-term employment relationship and emotional commitment between employees and their organization, helping enhance full understanding of and mutual respect for each other’s roles (Zhou and Li, 2015). In addition, the preceding hypothesis has demonstrated that high-quality relationships exert a significant positive effect on virtual team performance. Therefore, this study proposes that high-quality relationships are likely to have a mediating effect on the influence of a distributive justice climate on virtual team performance.

In addition, social interdependence theory points out that the social interdependence exists when the results of individuals and teams are influenced by their own actions and those of others (Johnson, 2009). In accordance with social interdependence theory, individuals are closely linked to the environment, and their behavior may be attributed to their own actions, combined with those of other individuals and the environment (Johnson, 2009). This theory does not apply to specific scenarios, but to all scenarios (Kelley et al., 2003). Existing studies have indicated that climate is a key issue in virtual teams (Martins et al., 2004). Therefore, distributive justice climate is particularly critical in virtual teams; it is conducive to solving many problems caused by the remote communication and collaboration of team members. The preceding hypothesis has demonstrated that distributive justice climate contributes to the development of high-quality relationships in virtual teams, and the establishment and maintenance of such relationships are important foundations of virtual team operation and management (Breuer et al., 2016). In accordance with social interdependence theory, this study infers that when individuals perceive a distributive justice climate in virtual teams, they interact with other members, establish and maintain high-quality relationships, and then improve virtual team performance. On this basis, the following hypothesis is presented:

*Hypothesis 3:* High-quality relationships mediate the relationship between distributive justice climate and virtual team performance.

#### Moderating effect of team proactive personality

Proactive personality refers to the behavior tendency of individuals to identify opportunities, take actions, and persevere long enough to bring about meaningful changes; it indicates that individuals will take a positive and spontaneous approach to accomplish tasks and achieve organizational goals (Li et al., 2014). Williams et al. (2010) believed that proactive personality studied at the individual level can also be adapted to active personality research at the team level. Given that team proactive personality is the primary approach to transforming individual level variables into team level variables (Mathieu et al., 2014), the current study analyzes the moderating effect of team proactive personality between distributive justice climate and high-quality relationships in virtual teams.

Restricted by technology dependence and the dispersion of members, virtual team members lack face-to-face communication, which is not conducive to timely problem solving and the cultivation of feelings among members (Purvanova, 2014). When team proactive personality is higher, team members generally have high proactive personality; members with high proactive personality will respond positively to the environment and exhibit proactive behavior to improve the *status quo* and ask questions (Williams et al., 2010). They also actively search for knowledge, information, and skills that are needed to better achieve their goals as individuals, teams, and organizations; moreover, they are willing to share these with other members (Parker and Collins, 2010). In addition, members with high proactive personality are more active in building effective social networks in teams to make other members and organizations support their ideas (Thompson, 2005). Therefore, virtual members with high proactive personalities will actively respond to distributive justice climate, strengthen coordination and harmonious interaction with other members, and then enhance the effect of distributive justice climate on high-quality relationships to a certain extent.

In contrast, virtual members with low proactive personalities tend to react passively to the environment, make fewer changes, fail to identify opportunities, and exhibit targeted behavior to cope with expectations (Zhao et al., 2010). Their avoidance of interaction with the environment will reduce or avoid communication and cooperation with other team members (Gong et al., 2012). Therefore, virtual members with low proactive personality do not take the initiative to identify distributive justice climate, affecting members’ attitude and behavior, and then weakening the effect of distributive justice climate on high-quality relationships. Therefore, the following hypothesis is proposed:

*Hypothesis 4:* Team proactive personality positively moderates the effect of distributive justice climate on high-quality relationships.

Given that team proactive personality is an important manifestation of team input affecting team output (Ali et al., 2021), this study proposes that the mediating effect of high-quality relationships is moderated by team proactive personality, and the whole model is a moderated mediation model. Specifically, high-quality relationships mediate the effect of a distributive justice climate on a virtual team’s performance, and their mediating effect depends on the level of team proactive personality. That is, when team proactive personality is at a high level, the indirect effect of a distributive justice climate affecting virtual team performance through high-quality relationships is enhanced. When team proactive personality is at a low level, the indirect effect of distributive justice climate affecting virtual team performance through high-quality relationships is weakened. Therefore, the following hypothesis is presented:

*Hypothesis 5:* Team proactive personality moderates the indirect effect of distributive justice climate on virtual team performance *via* high-quality relationships. This indirect effect is stronger when the team proactive personality is at a high level.

**FIGURE 1:**
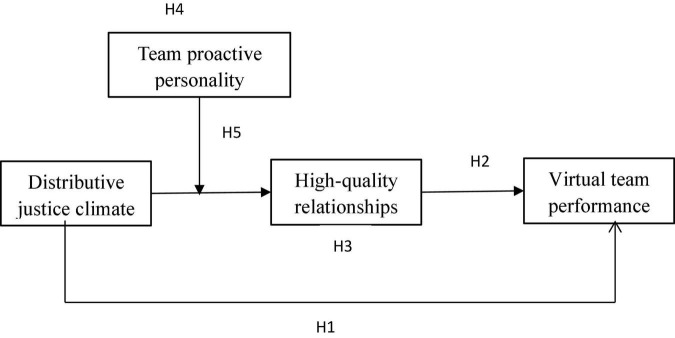
The proposed model.

The conceptual model of this study is illustrated in Figure 1.

## Method

### Participants and procedures

The study collected sample data from 327 virtual team members belonging to 75 teams from various enterprises located in Southwest China. The survey took the form of multiple questionnaires in two stages. In the first stage, the questionnaire included the basic information of members and teams. Distributive justice climate scale, proactive personality scale, and high-quality relationship scale were collected. In the second stage, the questionnaire included team performance. Before starting the survey, the investigators communicated with the personnel managers of different companies to identify the teams and members who finally participated in the questionnaire. A code was assigned to different teams, such that teams and members may be matched one by one after all the questionnaires were collected.

In the first stage, 486 questionnaires were distributed and 453 were recovered. In the second stage, 502 questionnaires were distributed and 413 were recovered. After matching valid questionnaires from the two rounds, 327 valid questionnaires from 75 teams were obtained. The average team size was 4.346, and the average team establishment year was 2.749. In the valid sample, males accounted for 46.5%, while females accounted for 53.5%. In terms of age, “25 years old and below” accounted for 14.4%, “greater than 25 and less than or equal to 35 years old” accounted for 69.1%, “greater than 36 and less than or equal to 45 years old” accounted for 16.6%, “greater than 46 and less than or equal to 55 years old” accounted for 4.3%, and “56 years old and above” accounted for 0.6%. In terms of education level, “junior college diploma and below” accounted for 14.7%, “undergraduate diploma” accounted for 66.4%, and “graduate diploma and above” accounted for 19.0%. In terms of work experience, “2 years of work experience or less” accounted for 11.3%, “3 to 5 years of work experience” accounted for 30.3%, “6 to 10 years of work experience” accounted for 36.7%, and “10 years of work experience or more” accounted for 21.7%. The basic information of all the participants is provided in Table 1.

**TABLE 1:** Basic information of sample (*N* = 327).

Items	Category	*N*	%	Items	Category	*N*	%
Gender	Male	152	46.5%	Education	Junior college and below	48	14.7%
	Female	175	53.5%		Undergraduate	217	66.4%
Age	0∼25	47	14.4%		Graduate and above	62	19.0%
	26∼35	160	49.1%	Tenure	0∼2 years	37	11.3%
	36∼45	54	16.6%		3∼5 years	99	30.3%
	46∼55	14	4.3%		6∼10 years	120	36.7%
	55 and above	2	0.6%		10 years and more	71	21.7%

### Measures

#### Distributive justice climate

The distributive justice climate scale, which has five scale items, was developed by Niehoff and Moorman (1993). An example of a scale item is “I think my salary level is fair.” In this study, Crobanch’s α was 0.824. Given that this study explored the distributive justice climate at the team level, the data of team members must be aggregated at the team level. We aggregate the perception level of individual employees on the distributive atmosphere of their team into the team level to represent the overall distributive atmosphere of the team. Through the aggregation analysis of distributive justice climate, Rwg, ICC (1), and ICC (2) were 0.841, 0.335, and 0.694, respectively. These values meet the requirements for team level data aggregation analysis.

#### High-quality relationships

High-quality relationships, which have 10 scale items, were measured using the scale developed by Gittell (2003). An example of a scale item is “In this enterprise, we have a common vision.” In this study, the Cronbach’s α of high-quality relationships was 0.826. Through the aggregation analysis of high-quality relationships, Rwg, ICC (1), and ICC (2) were 0.876, 0.336, and 0.695, respectively. These values meet the requirements for team level data aggregation analysis.

#### Proactive personality

The proactive personality scale, which has 10 scale items, was developed by Li et al. (2014). An example of a scale item is “Team members are constantly looking for new ways to improve my life.” In this study, the Cronbach’s α of proactive personality was 0.916. Using the mean or sum of individual characteristics (e.g., cognitive ability or personality) is the most appropriate approach when a task requires the knowledge, skills, and abilities of every team member (Chiu et al., 2016). Existing studies have widely used the method of measuring individual traits and then aggregating them to obtain team average values to measure team proactive personality (Williams et al., 2010). Therefore, through the aggregation analysis of the proactive personality of team members, Rwg, ICC (1), and ICC (2) were 0.935, 0.285, and 0.641, respectively. These values meet the requirements for team level data aggregation analysis.

#### Team performance

Team performance was measured using the scale developed by Gonzalez-Mule et al. (2014), which has four question items. An example of a question item is “The team has achieved high performance.” In this study, the Cronbach’s α of team performance was 0.887. Through the aggregation analysis of team performance, Rwg, ICC (1), and ICC (2) were 0.894, 0.342, and 0.701, respectively. These values meet the requirements for team level data aggregation analysis.

#### Control variables

Previous studies have shown that in the process of statistical analysis, team performance is affected by many team characteristics; among which, team size will affect team output to a certain extent (Zee and Weiss, 2019). Team establishment year reflects the accumulation of knowledge, experience, and methods in team management; it will affect the scale and structure of existing knowledge resources necessary for virtual team innovation and improvement (Brown et al., 2021). These team characteristics may affect virtual team performance. Therefore, team size and establishment year were selected as the control variables in this study to verify the relationship among key variables accurately and increase external validity in this study.

## Results

### Common method bias test

To avoid severe common methodological bias, this study adopted the suggestion of Podsakoff et al. (2003) and conducted a Harman single-factor test to investigate whether the results were disturbed by homologous method bias. Factor analysis was conducted on the scale items of distributive justice climate, proactive personality, and high-quality relationships reported by team members in the first stage. The test results showed that the total contribution rate was 71.305%. The variance interpretation rate of the first factor was 35.805%. No single factor was found, and no single factor in which the variance ratio was overwhelming was identified. Therefore, the data used in this study do not suffer from the common method bias.

Simultaneously, this study used the unmeasurable latent method factor test, which means that the load of all the measured constructs exists not only on the subordinate construct factor but also on the latent construct factor. In this study, the average variance extraction of the homologous error as a latent construct in the four-factor model was 19.407%, which was 25% below the criterion for determining whether orthologous variance could be regarded as a latent construct, indicating that the homologous variance could not be a latent variable that affects the variables in this study (Williams et al., 1989).

#### Confirmatory factor analysis

In this study, Amos 21.0 was used for confirmatory factor analysis (CFA) to analyze the discriminant and structural validities of the variables. The hypothesis model consisted of a four-factor model composed of a distributive justice climate, high-quality relationships, team proactive personality, and virtual team performance. Three competitive models were proposed simultaneously. The three-factor model combined distributive justice climate and team proactive personality into one factor. The two-factor model combined distributive justice climate and team proactive personality into one factor and high-quality relationships and virtual team performance into another factor. The one-factor model combined distributive justice climate, high-quality relationships, virtual team performance, and team proactive personality into one factor. The results are presented in Table 2. The fitting indexes supported the hypothetical four-factor model [χ^2^/*df* = 2.699, incremental fit index (IFI) = 0.956, Tucker–Lewis index (TLI) = 0.942, comparative fit index (CFI) = 0.956, and root-mean-square error of approximation (RMSEA) = 0.072], which achieved good discriminant validity.

**TABLE 2:** Confirmatory factor analysis.

Model	Factor	*χ^2^*	*Df*	*χ^2^/df*	*IFI*	*TLI*	*CFI*	*RMSEA*
Basic model	Four factors	159.215	59	2.699	0.956	0.942	0.956	0.072
Model 1	Three factors	405.774	62	6.545	0.850	0.810	0.849	0.130
Model 2	Two factors	475.250	64	7.426	0.821	0.780	0.820	0.140
Model 3	One factor	1015.022	65	15.616	0.586	0.500	0.583	0.212

#### Descriptive statistics

The mean, standard deviation, and correlation coefficient of each variable are provided in Table 3. Distributive justice climate was significantly and positively correlated with virtual team performance (*r* = 0.476, *p* < 0.01), while the variable “high-quality relationships” was significantly and positively correlated with virtual team performance (*r* = 0.560, *p* < 0.01). These results provide preliminary support for the proposed hypothesis.

**TABLE 3:** Means, standard deviations, and correlation coefficients between variables.

Variables	Mean	SD	1	2	3	4	5	6
1. Team size	4.346	1.672						
2. Team establishment years	2.749	0.646	−0.239*					
3. Distributive justice climate	5.252	0.610	−0.087	0.03				
4. Team proactive personality	5.368	0.470	−0.087	0.132	0.617**			
5. High-quality relationships	5.505	0.471	0.089	0.133	0.529**	0.491**		
6. Virtual team performance	5.508	0.592	−0.013	0.224	0.476**	0.463**	0.560**	

*p < 0.05, **p < 0.01.

#### Regression analysis

**FIGURE 2:**
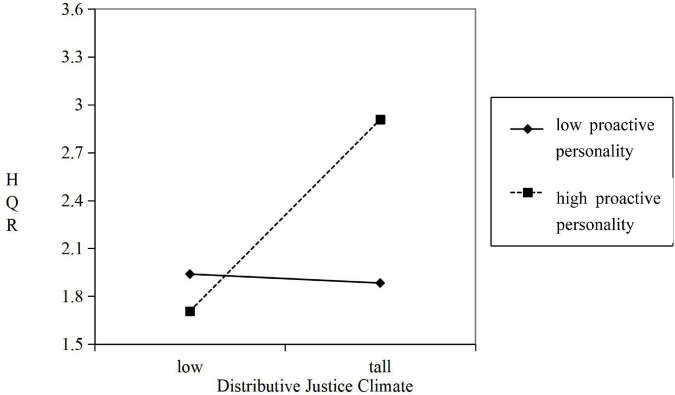
Moderated mediating effect of team proactive personality. HQR, high-quality relationships.

In this study, SPSS 22.0 was used to test the hypotheses. The regression results presented in Figure 2 show that the distributive justice climate exerted a significant positive effect on virtual team performance (Model 5, β = 0.462, *p* < 0.001). Thus, *Hypothesis 1* is supported. High-quality relationships exerted a significant positive effect on virtual team performance (Model 6, β = 0.758, *p* < 0.001). Thus, *Hypothesis 2* is supported.

To verify the mediating effect proposed in *Hypothesis 3*, distributive justice climate and high-quality relationships were inputted into the regression equation. Comparing Models 5 and 6, high-quality relationships still exerted a significant positive effect on virtual team performance (β = 0.556, *p* < 0.001), but the effect of distributive justice climate on virtual team performance remained significant when compared with Model 5 (β = 0.254, *p* < 0.05), indicating that high-quality relationships exerted a mediating effect between distributive justice climate and virtual team performance. Thus, *Hypothesis 3* is preliminary supported. We further used the bootstrapping method to detect the mediating effect. This study used 2,000 bootstrapping samples, with distributive justice climate as the independent variable, high-quality relationships as the mediator, and virtual team performance as the dependent variable. The indirect effect of a distributive justice climate on virtual team performance was at 95% confidence interval (CI) [0.083, 0.389], CI excluded 0, and the indirect effect was significant. Thus, *Hypothesis 3* was supported again.

Through stratification regression analysis, the moderating effect of team proactive personality on distributive justice climate and high-quality relationships behavior was verified. In Model 3 of Table 4, the interaction of distributive justice climate and team proactive personality exerted a significant positive effect on high-quality relationships (β = 0.235, *p* < 0.05); that is, the higher the team proactive personality, the stronger the positive effect of distributive justice climate on high-quality relationships. Thus, *Hypothesis 4* is supported. We adopted the methods proposed by Aiken and West (1991) to clearly demonstrate the moderating effect of a team proactive personality. We drew the moderating effect diagram in accordance with the mean standard deviation of team proactive personality. As shown in Figure 2, when the level of team proactive personality was low (mean minus one standard deviation), the effect of distributive justice climate on high-quality relationships was negative but insignificant (β = −0.028, *p* > 0.05). When the level of team proactive personality was high (mean plus one standard deviation), distributive justice climate exerted a significant positive effect on high-quality relationships (β = 0.600, *p* < 0.001). A significant difference existed between high and low groups (β = 0.628, *p* < 0.05).

**TABLE 4:** Regression analysis results.

	High-quality relationships			Virtual team performance			
	Model 1	Model 2	Model 3	Model 4	Model 5	Model 6	Model 7
Team size	0.032	0.044	0.050*	0.015	0.029	−0.009	0.005
Team establishment years	0.107	0.104	0.098	0.214	0.210*	0.133	0.153
Distributive justice climate		0.375***	0.286***		0.462***		0.254*
Team proactive personality			0.198				
Interactive items			0.235*				
High-quality relationships						0.758***	0.556***
*R* ^2^	0.033	0.322***	0.397***	0.052	0.277***	0.337***	0.385***
*ΔR^2^*	0.033	0.289***	0.075*	0.052	0.225***	0.285***	0.048*
*F*	1.229	11.238***	9.076***	1.974	9.085***	12.042***	10.950***

*p < 0.05, ***p < 0.001.

The regression coefficients in the table are non-standardized coefficients; Interaction item: team distributive justice climate × team proactive personality.

*Hypothesis 5* was tested in accordance with the method proposed by Edwards and Lambert (2007). First, the samples were divided into two groups in accordance with the standard deviation of the mean of the moderating variable minus or plus one standard deviation. Then, the size and difference of the two indirect effects were calculated for 2,000 bootstrapping samples. If the difference between the indirect effects of the two groups was significant, then moderated mediation was established. We tested moderated mediation by using Mplus 7.0 software (Muthen & Muthen). As indicated in Table 5, when the level of team proactive personality was high, the indirect effect of distributive justice climate on virtual team performance was significant (mediation effect value = 0.209, *p* < 0.05, 95% CI did not contain 0 [0.080, 0.419]). When the level of team proactive personality was low, the indirect effect of distributive justice climate on virtual team performance was insignificant (indirect effect value = 0.100, *p* > 0.05, 95% CI contained 0 [−0.015, 0.274]). The difference was significant (indirect effect value = 0.109, *p* < 0.05), and 95% CI did not contain 0 [0.028, 0.287], showing that team proactive personality positively moderated the indirect effect of distributive justice climate on virtual team performance. Thus, the moderated mediation of *Hypothesis 5* is supported.

**TABLE 5:** Results of the moderated mediation model.

	Distributive justice climate (X) → High-quality relationships (M) → Virtual team performance (Y)			
	Stage I (P_MX_)	Stage II (P_YM_)	Indirect effect (P_MX_ × P_YM_)	Indirect effect of 95% The confidence interval
High team proactive personality	0.355***	0.590*	0.209*	[0.080, 0.419]
Low team proactive personality	0.170*	0.590*	0.100	[−0.015, 0.274]
Difference	0.185*	0	0.109*	[0.028, 0.287]

*p < 0.05, ***p < 0.001.

## Discussion

This study builds a moderated mediation model. That is, it examines the mediating effect of high-quality relationships between distributive justice climate and virtual team performance, the moderating effect of team proactive personality between distributive justice climate and high-quality relationships, and the indirect effect of distributive justice climate on virtual team performance through high-quality relationships. On the basis of data from 327 virtual team members from 75 high-tech enterprises in Southwest China, the proposed research hypothesis is supported.

### Research conclusion

(1)In accordance with social interdependence theory, this study explores the effect of distributive justice climate on virtual team performance. At present, the mediating effect between distributive justice climate and virtual team performance has not yet been clearly studied and empirically tested, and relevant research is still in its infancy. Therefore, this study solves this problem by constructing and empirically verifying the effect model of “distributive justice climate → high-quality relationships → virtual team performance.”

(2)This study explores team proactive personality as the primary boundary condition. Virtual team members, who are scattered in different regions, are restricted by technology dependence, their communication lacks the characteristics of mutual contact, and the transmission and processing of information may be delayed or misunderstood (Purvanova, 2014). Therefore, the effect of a team proactive personality on timely problem solving and the cultivation of members’ feelings is becoming increasingly important. However, only a few empirical studies have been conducted on team proactive personality, mostly at the level of theoretical description and discussion (Grant et al., 2011; Bradley et al., 2013). Therefore, this study focuses on a virtual team. It discusses the moderating effect of team proactive personality between distributive justice climate and high-quality relationships and the indirect effect of distributive justice climate on virtual team performance through high-quality relationships, making it a useful supplement to relevant research.

### Practical implications

(1)To promote virtual team performance, the primary task of virtual team managers is to create and maintain a distributive justice climate. On the one hand, we should unify, open, and make transparent the distribution standards of team resources and rewards and try to let all members agree to them. Only by adopting the same standards can we make the distribution results (including salary, incentive measures, and team honor sharing) sufficiently fair and equal, and thus, lay the foundation for creating a distributive justice climate. On the other hand, we should widely solicit the opinions of team members and use scientific statistical methods to unify standards, such that members can form a common view on the distribution standards of team resources and rewards, further maintaining a distributive justice climate in a virtual team.

(2)Virtual team managers should give attention to the critical role of high-quality relationships in improving team performance. First, from the perspective of team vision, individual and team goals should be coordinated to form a consistent vision and team commitment, enhancing the team identity of virtual team members. Second, in terms of shared knowledge, virtual team members should be encouraged to share knowledge, information, and resources to strengthen communication among members, and thus, improve virtual team performance. Finally, in terms of mutual respect, virtual team members are encouraged to expand their interpersonal network, enhance interpersonal mutual trust, and promote team harmony to improve virtual team performance.

(3)Virtual team managers must give attention to and investigate proactive personality traits at the team level in the practice of human resource management. Specifically, virtual team managers may set the personal characteristics of proactive personality as the broader criteria for selecting and training members. When managers need to build a new virtual team or recruit new members, proactive personality tests should be added to understand the candidates, and managers should focus on the proactive personality traits of team members if they plan to promote a team member. Furthermore, the importance of emphasizing team proactive personality should be incorporated into the daily training of virtual team members. The proactive personality of virtual team members will be shaped through consciousness training and situational exercises to improve the average level of team proactive personality.

### Limitations and future directions

This study exhibits the following limitations. First, team performance in this study is self-reported, which may exhibit the problem of common method bias. Although some techniques have been used to reduce the risk of common method bias, CFA has also been performed to ensure the reliability of the research results. In future research, quantitative methods of performance evaluation, such as objective management, important event, and relative comparison methods, may be used to formulate objective indicators to evaluate virtual team performance.

Second, the sample of this study is only limited to the virtual teams of high-tech enterprises in Southwest China, affecting the universality of the conclusion to a certain extent. Future research may expand the sample scope.

Third, this study is conducted in China, and the Chinese people are more sensitive to the distributive justice climate. In view of the effect of cultural differences on individual attitude and behavior, the conclusion may have limitations. Future research may consider the use of transnational samples. A more comprehensive study may be conducted on the effect of distributive justice climate on virtual team performance in different cultural backgrounds.

## Data availability statement

The raw data supporting the conclusions of this article will be made available by the authors, without undue reservation.

## Ethics statement

Ethical review and approval was not required for the study on human participants in accordance with the local legislation and institutional requirements. The participants provided their written informed consent to participate in this study in accordance with the Declaration of Helsinki. Research respondents were ensured confidentiality and anonymity. All participation was voluntary.

## Author contributions

BH wrote the manuscript under the guidance of XY. XY contributed to the study design and critical revisions. ML was in charge of data analysis. AW was in charge of language polishing. YY was in charge of manuscript proofreading and checking. All authors contributed to the article and approved the submitted version.
